# Protective Effects of Valproic Acid, a Histone Deacetylase Inhibitor, against Hyperoxic Lung Injury in a Neonatal Rat Model

**DOI:** 10.1371/journal.pone.0126028

**Published:** 2015-05-04

**Authors:** Merih Cetinkaya, Mehmet Cansev, Ferhat Cekmez, Cuneyt Tayman, Fuat Emre Canpolat, Ilker Mustafa Kafa, Esra Orenlili Yaylagul, Boris W. Kramer, Serdar Umit Sarici

**Affiliations:** 1 Gulhane Military Medical Academy, Department of Pediatrics, Division of Neonatology, Ankara, Turkey; 2 Uludag University Medical School, Department of Pharmacology, Bursa, Turkey; 3 Uludag University Medical School, Department of Anatomy, Bursa, Turkey; 4 Uludag University School of Arts and Sciences, Department of Biology, Bursa, Turkey; 5 Maastricht University Medical Center, Department of Pediatrics, Maastricht, Netherlands; The Ohio State Unversity, UNITED STATES

## Abstract

**Objective:**

Histone acetylation and deacetylation may play a role in the pathogenesis of inflammatory lung diseases. We evaluated the preventive effect of valproic acid (VPA), a histone deacetylase (HDAC) inhibitor, on neonatal hyperoxic lung injury.

**Methods:**

Forty newborn rat pups were randomized in normoxia, normoxia+VPA, hyperoxia and hyperoxia+VPA groups. Pups in the normoxia and normoxia+VPA groups were kept in room air and received daily saline and VPA (30 mg/kg) injections, respectively, while those in hyperoxia and hyperoxia+VPA groups were exposed to 95% O_2_ and received daily saline and VPA (30 mg/kg) injections for 10 days, respectively. Growth, histopathological, biochemical and molecular biological indicators of lung injury, apoptosis, inflammation, fibrosis and histone acetylation were evaluated.

**Results:**

VPA treatment during hyperoxia significantly improved weight gain, histopathologic grade, radial alveolar count and lamellar body membrane protein expression, while it decreased number of TUNEL(+) cells and active Caspase-3 expression. Expressions of TGFβ3 and phospho-SMAD2 proteins and levels of tissue proinflammatory cytokines as well as lipid peroxidation biomarkers were reduced, while anti-oxidative enzyme activities were enhanced by VPA treatment. VPA administration also reduced HDAC activity while increasing acetylated H3 and H4 protein expressions.

**Conclusions:**

The present study shows for the first time that VPA treatment ameliorates lung damage in a neonatal rat model of hyperoxic lung injury. The preventive effect of VPA involves HDAC inhibition.

## Introduction

Bronchopulmonary dysplasia (BPD) is a chronic lung disease developing especially in premature infants with significant morbidity and mortality [1]. The pathogenesis of BPD is multifactorial resulting in chronic inflammation of the airways that results in a simplified lung structure [2]. To date, despite antenatal steroids, vitamin A, and caffeine being suggested as beneficial agents for BPD prevention, BPD is still the most common adverse outcome after preterm birth and the use of post-natal steroids as potent anti-inflammatory drugs is still under study [1,3,4]. Recent evidence suggests a net benefit of postnatal glucocorticoid therapy when administered shortly after the first week of life to premature infants with early and persistent pulmonary dysfunction [5]. Similarly, in a recent Cochrane review, late steroid therapy after 7 days of life for chronic lung disease was suggested to reduce neonatal mortality without significantly increasing the risk of adverse long-term neurodevelopmental outcomes [6].Therefore, it is necessary to develop new strategies for the prevention of BPD.

Chronic inflammation can be maintained by different triggers which promote transcription of pro-inflammatory cytokines. Transcription factors target gene transcription after activation and recruit transcriptional co-activators and chromatin-remodeling enzymes that allows subsequent inflammatory gene expression [7]. The transcription of genes is mainly made possible by rearrangement of the chromatin structure by histone methylation and deacetylation, which are all epigenetic modification [8]. Epigenetic regulation of inflammation has therefore several components. Changes in histone acetylation are involved in induction of pro-inflammatory genes in human lung cells. Histone acetyltransferases (HATs) are expressed and activated in an abnormal fashion in inflammatory diseases and, histone deacetylase (HDAC) inhibitors show anti-inflammatory effects [9]. Differentially-regulated chromatin remodeling pathways were reported in umbilical cord samples of infants who developed BPD by altering HDAC ratio, resulting in histone hypoacetylation. This suggests the use of HDAC inhibitors for the prevention of BPD development [10]. However, current therapies of BPD do not modulate the epigenetic compound of chronic inflammation.

A candidate drug for this purpose is Valproic acid (VPA), an HDAC inhibitor that is clinically used as an anti-epileptic and mood-stabilizing agent. VPA is an HDAC inhibitor of class I, as well as class II histone deacetylases [11] which results in anti-inflammatory properties by reducing transcription of pro-inflammatory cytokines [12,13]. This effect was shown in a lipopolysaccharide (LPS)-induced septic shock model where VPA attenuated multiple organ dysfunctions by ameliorating histopathological lung injury, preventing pulmonary inflammation by reversing the reduction in histone H3 acetylation in lung tissue [14].

However, to the best of our knowledge, no experimental study has investigated VPA’s effectiveness in neonatal hyperoxic lung injury. Therefore, the aim of this study was to evaluate the possible preventive effect of VPA in a neonatal rat model of hyperoxic lung injury and the involvement of HDAC inhibition in VPA’s effect. We used a rat model in which neonatal exposure to hyperoxia induced lung simplification resembling the lung structure of patients with BPD.

## Material and Methods

### Animals and experimental design

The study was approved by Experimental Animal Ethics Committee of Gulhane Military Medical Academy (Ankara, Turkey, Permit number: 2011–6) and the experiments conformed to the National Institutes of Health Guide for the Care and Use of Laboratory Animals (NIH Publications No. 80–23) revised 1996 and EC Directive 86/609/EEC.

Sprague-Dawley rats with dated pregnancies were housed in individual cages with free access to water and food. Pups born with spontaneous delivery to Sprague-Dawley pregnant rats were pooled, randomized, and delivered back to nursing dams. The offsprings from one dam were randomly introduced into all four study groups. A total of 40 pups born to 4 dams were divided into 4 groups from each dam as follows: normoxia group (subjected to room air containing 21% O_2_ and received saline), normoxia+VPA group (subjected to room air containing 21% O_2_ and received VPA), hyperoxia group (subjected to 95% O_2_ and received saline), and hyperoxia+VPA group (subjected to 95% O_2_ and received VPA).

Experiments began immediately after birth and continued throughout P10 as described previously [15]. Nursing dams were rotated between hyperoxia and room air-exposed pups every 24h to prevent oxygen toxicity. In pups subjected to hyperoxia, continuous 95% O_2_ exposure was achieved in a Plexiglas chamber (70x60x30 cm) by a flow-through system. The oxygen level inside the Plexiglas chamber was monitored continuously with a Ceramatec (MAXO2) oxygen analyzer. Carbondioxide (CO_2_) concentration was kept below 0,5% using a gas monitor (Apex, BW Technologies, Lincolnshire, IL). Temperature and humidity were maintained at 22°C-25°C and 60%-70%, respectively.

Intraperitoneal (i.p.) injections of saline (4 ml/kg) in normoxia and hyperoxia groups and those of VPA (30 mg/kg; Sigma-Aldrich, St. Louis, MO) in hyperoxia+VPA group were performed daily from the 1st day of life (P1) throughout P10. Pups in each group were weighed daily and weights were recorded.

### Lung Tissue Preparation

Pups were sacrificed at P10 under deep anesthesia and all efforts were performed to minimize suffering. Right lungs of rat pups were excised and snap frozen for analyzing tissue cytokines; HDAC activity; expressions of acetylated H3 and H4, TGFβ3, phospho-SMAD2 and active Caspase-3; as well as oxidant/antioxidant enzyme activities and MDA content, while left lungs were perfused for histopathologic and immunohistochemical evaluation after ligation of the right main bronchus.

### Histopathologic and Immunohistochemical Evaluation of the Lungs

Left lungs were fixed by perfusion with 0.1 M phosphate buffered saline (PBS; pH 7.4) containing 4% paraformaldehyde (PFA). Trachea was ligated with a surgical suture, and lungs were incubated in fresh 4% PFA-PBS solution on ice for 4–5 h. The lungs were paraffin-embedded for obtaining 5 μm sections which were then mounted onto poly-L-lysine-coated slides (Paul Marienfeld GmbH&Co., Lauda-Konigshofen, Germany), stained with standard haematoxylin-eosin and Masson’s trichrome techniques for histopathologic evaluations and with ABC technique for lamellar body membrane protein (LBMP) expression as described previously [15–17].

Apoptosis was evaluated by Terminal Deoxynucleotidyl Transferase dUTP Nick End Labeling (TUNEL) technique using in situ cell death detection POD kit (Roche Molecular Biochemicals, Mannheim, Germany) as described previously [15].

### Western Blot Analyses

TGFβ isoforms (β1, β2, β3) play a role in normal tissue repair following lung injury [18]. The intracellular signaling pathway of TGFβ receptors is mediated by a family of transcription factors, called SMAD proteins. The receptor-regulated SMAD2 and/or SMAD3, in combination with SMAD4 positively regulate the effects of TGFβ [18]. Therefore, we also evaluated the effect of VPA treatment on TGFβ pathway and SMAD protein expression in this hyperoxic lung injury model. In addition, members of Bcl-2 gene family are known as the key regulators of cell survival, apoptosis and necrosis and bcl-2 represents an anti-apoptotic protein [19]. Right lungs were homogenized in ice-cold PBS and aliquoted homogenates were used for total protein analysis by bicinchoninic acid (BCA) assay (Thermo Fisher Scientific, Rockford, IL). Aliquots of homogenates were used for determining specific proteins using antibodies against active Caspase-3 (Abcam, Cambridge, MA), bcl-2 (Cell Signaling Technology, Danvers, MA), TGFβ1 (Thermo Scientific, Rockford, IL), TGFβ3 (Thermo Scientific, Rockford, IL), phospho-SMAD2 (pSer465/467; Thermo Scientific, Rockford, IL), acetyl-histone H3 (Cell Signaling Technology, Danvers, MA) and acetyl-histone H4 (Millipore, Billerica, MA) as described previously [15]. Equal protein loading was confirmed by incubating the stripped membranes with structural protein β-actin (Abcam, Cambridge, MA).

### HDAC Activity

HDAC activity was assayed using a colorimetric detection kit (Upstate, Temecula, CA) according to the manufacturer’s instructions. Absorbances were detected at 405 nm and results were expressed as the percentage of Normoxia group.

### Biochemical analyses

Cell-free supernatants of lung tissue homogenates were used for determinig lung tissue pro-inflammatory cytokine (TNF-α, IL-6 and IL-1β) contents by specific enzyme-linked immunosorbent assay (ELISA) kits (R&D Systems, Minneapolis, MN) [17].

Activities of superoxide dismutase (SOD), glutathione peroxidase (GSH-Px) and myeloperoxidase (MPO), as well as malondialdehyde (MDA) content of lung tissues were measured by spectrophotometric (UV-1700, Shimadzu, Japan) analyses [15].

### Statistics

Statistical analyses were performed using SPSS 16.0 software (IBM Corporation, Armonk, NY). Data were expressed as mean±standard error of means (SEM). Normal distributions of data were graphically examined with Shapiro-Wilk test. Categorical variables were compared with Chi square test. Immunohistochemical scores were assessed by Kruskal Wallis test. Treatment groups were compared using One-Way ANOVA and significance was determined using post-hoc Tukey test or Bonferroni’s correction for multiple comparisons where applicable. p<0.05 was considered statistically significant.

## Results

**Fig 1:**
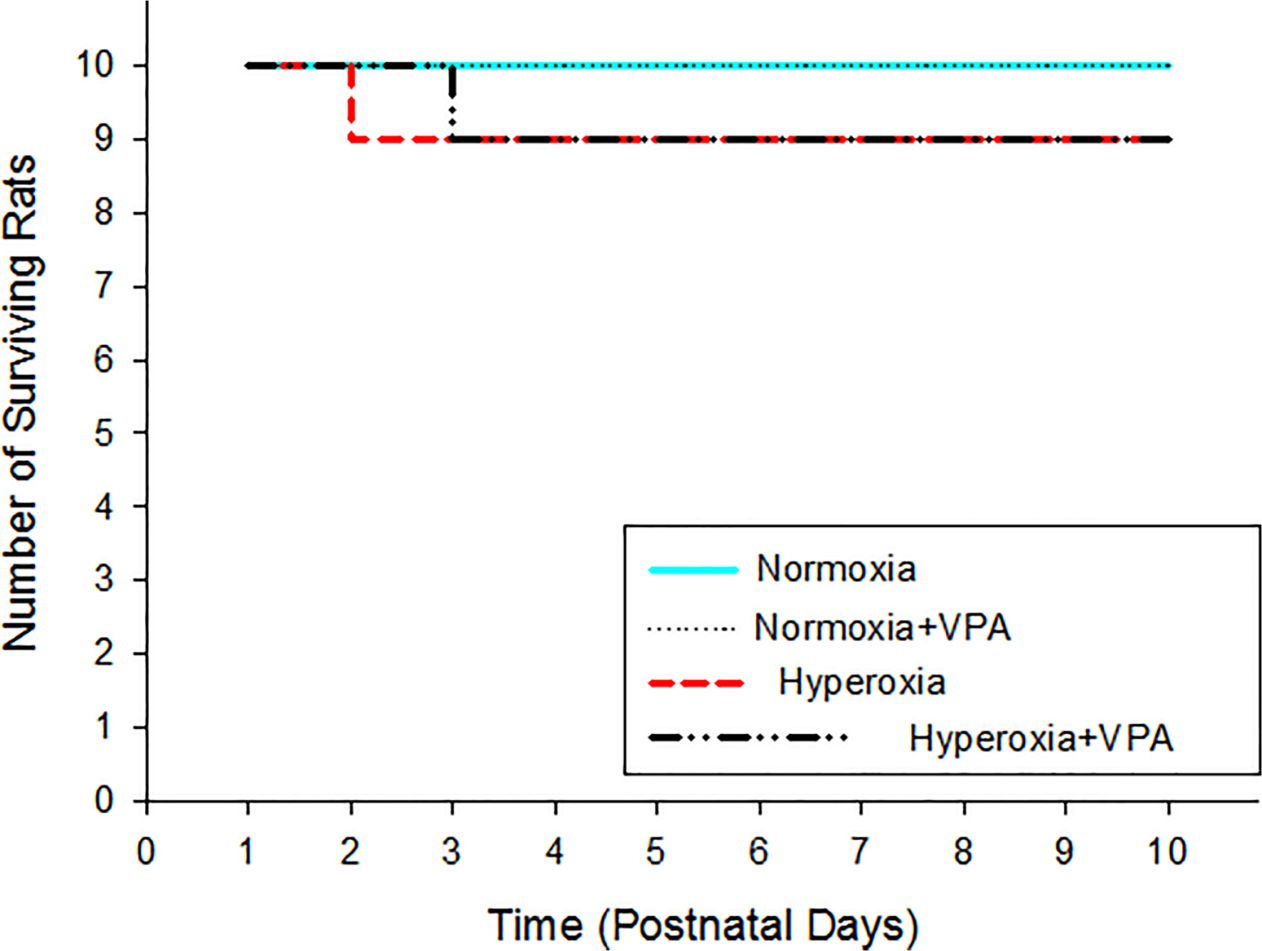
Number of surviving rat pups in experimental groups.

**Fig 2:**
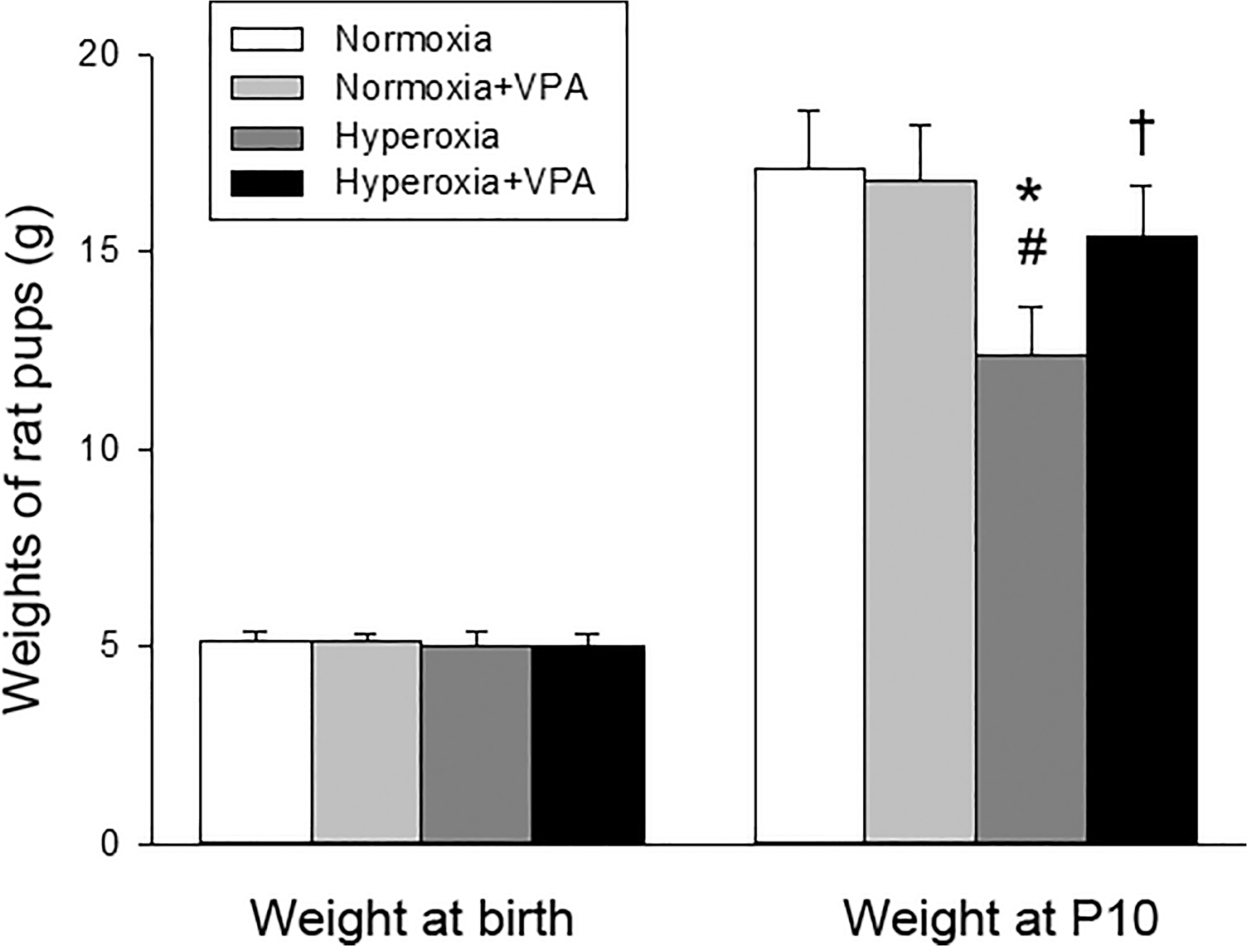
Bar graph depicting body weights of rat pups at birth and P10. *p<0.05 compared to Normoxia group; #p<0.05 compared to Normoxia+VPA group; and †p<0.05 compared to Hyperoxia group using One-Way ANOVA followed by post-hoc Tukey test.

We started the experiment with 10 rat pups in each group. No pups died in Normoxia or Normoxia+VPA groups throughout the study period. On the other hand, one pup in each Hyperoxia or Hyperoxia+VPA group died on the 2^nd^ or the 3^rd^ postnatal days, respectively. No significant difference was found in terms of survival rate among experimental groups (p>0.05) and our analyses were not affected by the number of surviving pups. A survival curve has been presented in Fig 1. The well-being of the rat pups was, in part, assessed with the weight gain during the experiment. Mean birth weights of pups in Normoxia, Normoxia+VPA, Hyperoxia and Hyperoxia+VPA groups (5.1±0.3 g vs. 5.1±0.2g vs. 5.0±0.4 g vs. 5.0±0.3 g, respectively) did not differ significantly. At the end of the experiment, the mean body weight of pups in Hyperoxia group (12.4±1.2 g) was significantly (p<0.05) lower than that of pups in Normoxia (17.1±1.5 g), Normoxia+VPA (16.8±1.4 g) and Hyperoxia+VPA (15.4±1.3 g) groups (Fig 2). The weight of the Hyperoxia+VPA group was not different to the Normoxia and Normoxia+VPA group.

**Fig 3:**
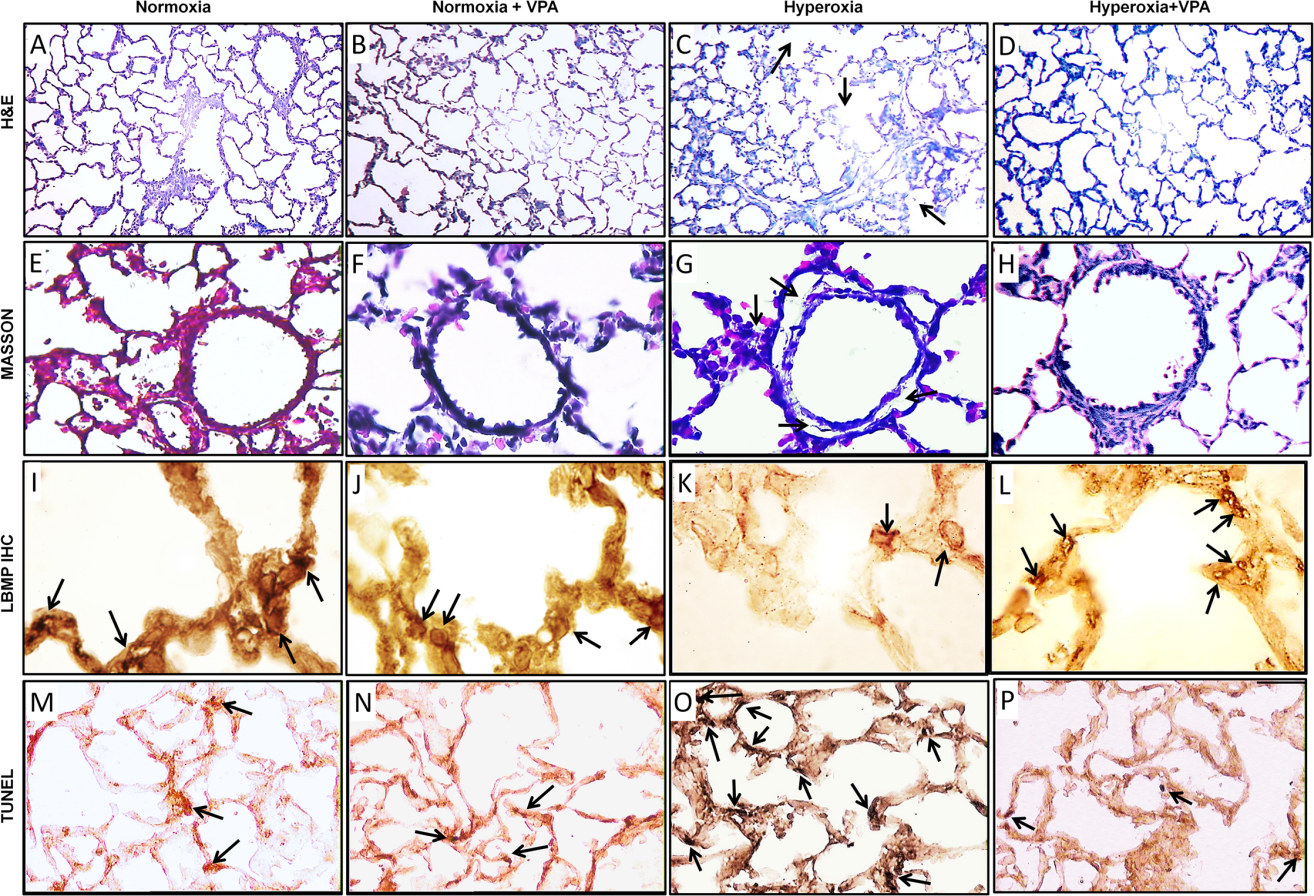
Histological examination of lung tissues by Haematoxylin-eosin (A-D, 100X magnification), Masson’s trichrome (E-H, 400X magnification), lamellar body membrane protein (LBMP) (I-L, 1000X magnification), and TUNEL-DAB stainings (M-P, 200X magnification) for Normoxia, Normoxia+VPA, Hyperoxia and Hyperoxia+VPA groups. Representative images show severe alveolar damage (panel C), cell infiltration and edema (panel G, arrow) in Hyperoxia group. Thickening of the alveolar septi or cell infiltration was not observed in Normoxia, Normoxia+VPA and Hyperoxia+VPA groups and panels A, B, D, E, F and H shows healthier and intact lung parenchymal appearance compared to hyperoxia group. Black arrows indicate positive immunoreactivity for LBMP and TUNEL(+) cells in panels I-P.

**Fig 4:**
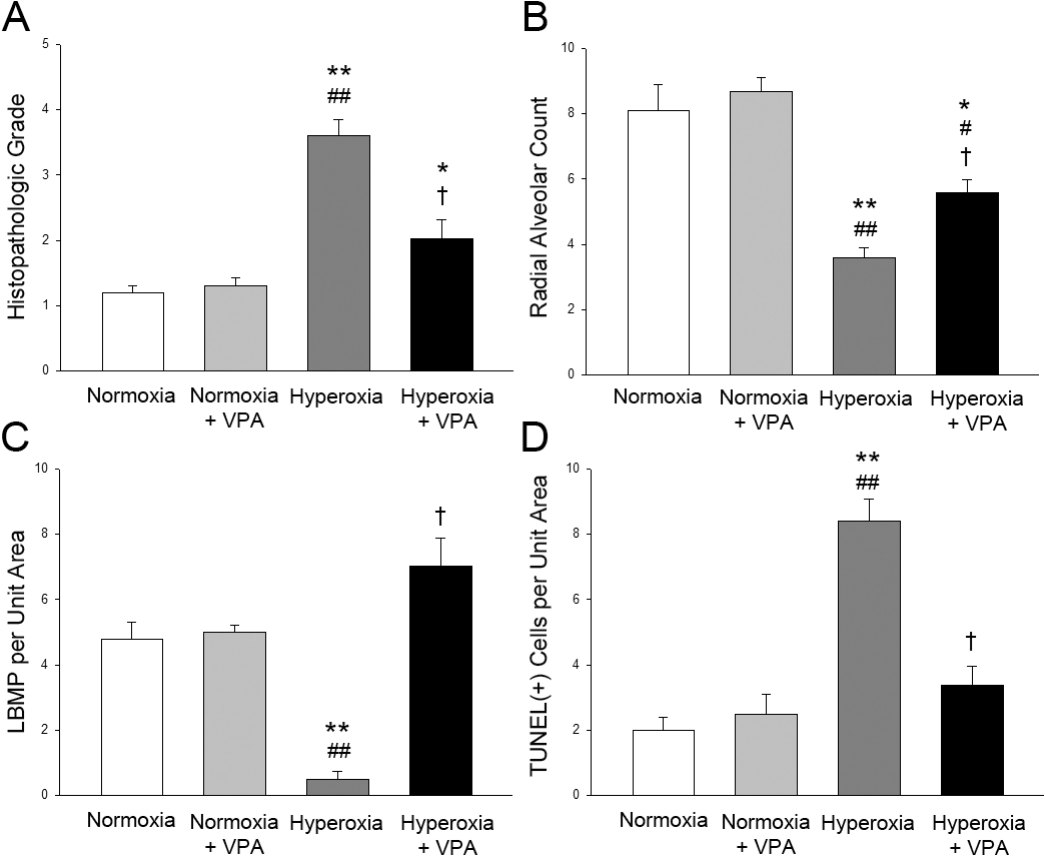
Bar graphs depicting Histopathologic Grade (A), Radial Alveolar Count (B), LBMP per Unit Area (C) and TUNEL(+) Cells per Unit Area (D) in lung tissues of rat pups. *p<0.05 and **p<0.001 compared to Normoxia group; #p<0.05 and ##p<0.001 compared to Normoxia+VPA group; and †p<0.05 and ††p<0.001 compared to Hyperoxia group using One-Way ANOVA followed by post-hoc Tukey test.

**Fig 5:**
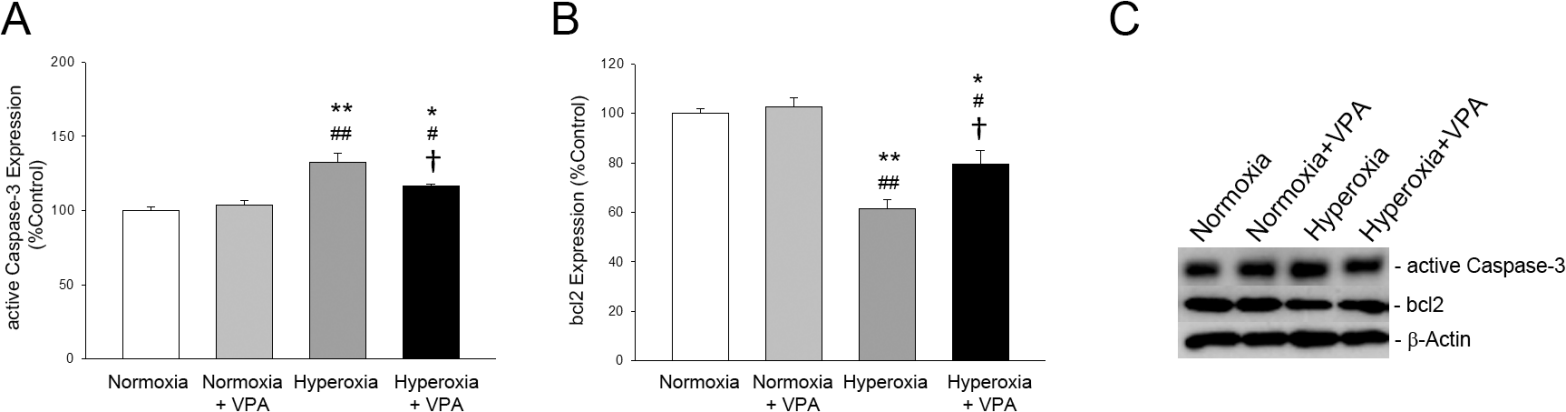
Bar graphs depicting active Caspase-3 (A) and bcl2 (B) expressions in lung tissues of rat pups. Panel C depicts representative bands for each protein, including β-Actin, the protein which was used as a loading control for western blotting. *p<0.05 and **p<0.001 compared to Normoxia group; #p<0.05 and ##p<0.001 compared to Normoxia+VPA group; and †p<0.05 compared to Hyperoxia group using One-Way ANOVA followed by post-hoc Tukey test.

The structure of the lung was assessed by histological examination. The mean histopathologic grade of lung injury in pups in Hyperoxia group was significantly (p<0.001) greater than that in Normoxia and Normoxia+VPA groups; VPA treatment significantly improved histological grading of lung injury compared with Hyperoxia group (Figs 3 & 4A). Thickening of the alveolar septi or cell infiltration was not observed in Normoxia, Normoxia+ VPA or Hyperoxia+VPA groups but in the Hyperoxia group (Fig 3). Masson’s trichrome-stained sections showed cell infiltration, edema and fibrosis in the Hyperoxia group but not in Normoxia, Normoxia+VPA and Hyperoxia+VPA groups (Fig 3). Mean radial alveolar count, reflecting the number of intact alveoli, was significantly (p<0.001) decreased in Hyperoxia group compared with Normoxia and Normoxia+VPA groups, while it was significantly greater in Hyperoxia+VPA group (p<0.05) compared to Hyperoxia group (Figs 3 & 4B). Similarly, the decrease in mean LBMP expression (p<0.001) in Hyperoxia group was significantly recovered by VPA treatment (p<0.001) (Figs 3 & 4C). The remodeling of the injured lung was assessed by evaluation of apoptosis including the number of apoptotic cells as well as expressions of active caspase-3 and bcl-2. When compared with the Normoxia and Normoxia+VPA groups, number of TUNEL(+) cells (Figs 3 & 4D) and active Caspase-3 expression (Fig 5A) were significantly increased in Hyperoxia group (p<0.001 and p<0.001, respectively) while VPA treatment significantly decreased number of TUNEL(+) cells (p<0.001) (Figs 3 & 4D) and active Caspase-3 expression (p<0.05) (Fig 5A). In contrast to active Caspase-3, levels of bcl-2, an anti-apoptotic protein, were significantly decreased in Hyperoxia group (p<0.001) compared with the Normoxia and Normoxia+VPA groups, while bcl-2 was significantly increased in Hyperoxia+VPA group in comparison with Hyperoxia group (p<0.05) (Fig 5B).

**Fig 6:**
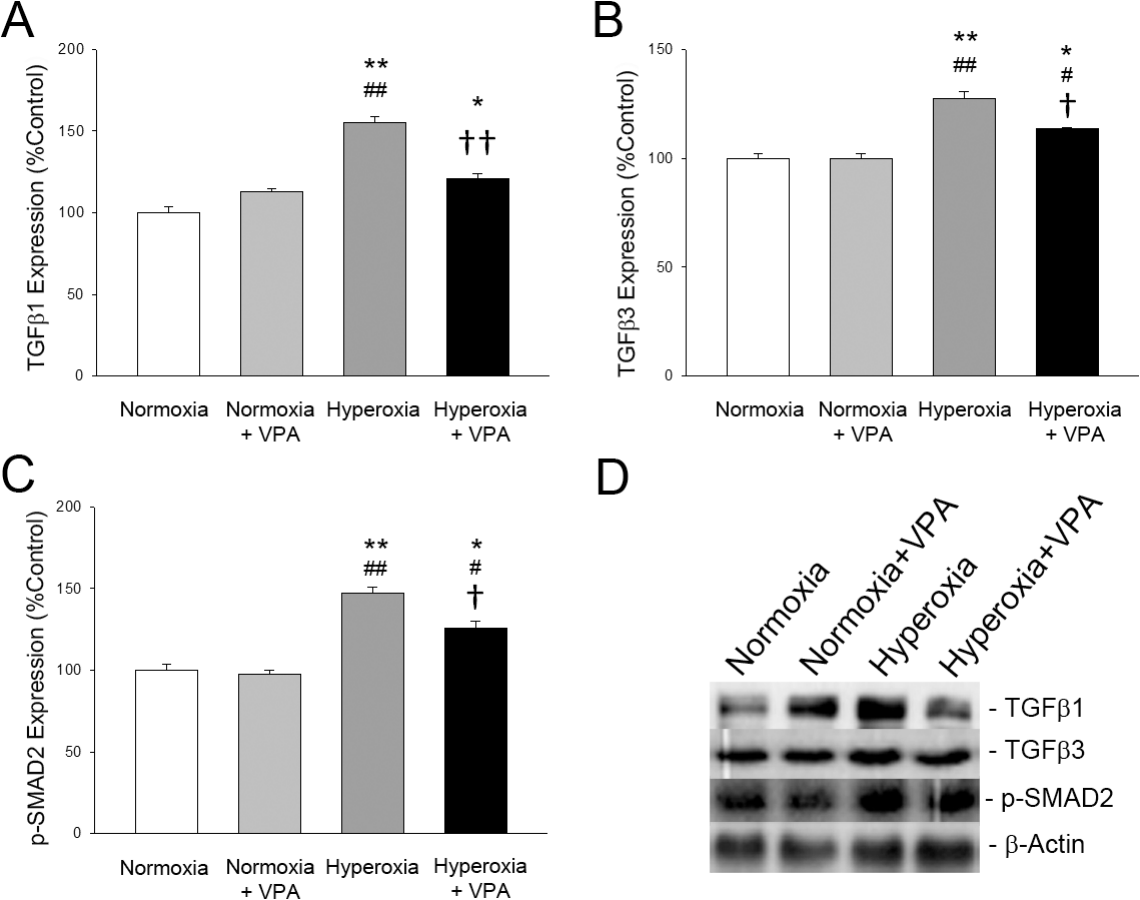
Bar graphs depicting TGFβ1 (A), TGFβ3 (B) and phospho-SMAD2 (C) expressions in lung tissues of rat pups. Panel D depicts representative bands for each protein, including β-Actin, the protein which was used as a loading control for western blotting. *p<0.05 and **p<0.001 compared to Normoxia group; #p<0.05 and ##p<0.001 compared to Normoxia+VPA group; and †p<0.05 and ††p<0.001 compared to Hyperoxia group using One-Way ANOVA followed by post-hoc Tukey test.

Inflammation was assessed by levels of IL-1β, IL-6, TNF-α and by MPO activity as a marker of neutrophil invasion. Levels of IL-1β, IL-6 and TNF-α in the lung tissues were increased in Hyperoxia group compared with the Normoxia and Normoxia+VPA groups (p<0.05 for all), whereas VPA treatment significantly reduced hyperoxia-induced elevations of IL-1β, IL-6 and TNF-α (p<0.05 for all) (Table 1). MPO activity was found to be increased by hyperoxia, whereas VPA treatment decreased it in hyperoxia (Table 1). Expressions of TGFβ1 and TGFβ3 (Fig 6A & 6B) and phospho-SMAD2 (Fig 6C) in Hyperoxia group were significantly (p<0.05) greater compared with Normoxia and Normoxia+VPA groups, whereas expressions of both proteins were reduced by VPA treatment (p<0.05).

**Table 1:** Comparison of lung tissue pro-inflammatory cytokine levels and superoxide dismutase (SOD), glutathione peroxidase (GSH-Px) and myeloperoxdase (MPO) activities and malonedialdehyde (MDA) content in all groups.

	Normoxia Group	Normoxia+VPA Group	Hyperoxia Group	Hyperoxia+VPA Group
**Cytokine Levels (pg/ml)**				
IL-1β	86.2 ± 6.4	89.4 ± 7.6	129.2 ± 10.1*	98.6 ± 7.5^†^
IL-6	36.1 ± 5.7	37.3 ± 5.4	58.4 ± 5.1*	42.5 ± 4.7^†^
TNF-α	101.6 ± 18.4	106.7 ± 12.6	242.5 ± 32.9*	141.7 ± 19.2^†^
**Biochemical Analyses**				
GSH-Px (U/mg protein)	13.2 ± 1.4	12.4 ± 1.2	4.1 ± 1.4*	9.5 ± 1.3^†^
SOD (U/mg protein)	115.8 ± 10.6	118.6 ± 9.1	60.5 ± 3.1**	90.8 ± 8.4^‡^
MPO (U/g protein)	25.7 ± 3.6	31.7 ± 4.4	82.7 ± 4.3**	56.5 ± 5.2^‡^
MDA (nmol/g protein)	24.6 ± 3.7	26.8 ± 4.1	80.4 ± 4.7**	47.6 ± 4.1^†^

*p<0,05 and

**p<0,01 compared to Normoxia group; and

^†^p<0,05 and

^‡^p<0,01 compared to Hyperoxia group using One-Way ANOVA followed by post-hoc Tukey test.

The maturity of the antioxidant enzyme systems was assessed by oxidant and antioxidant enzyme activities. The decreases in lung tissue GSH-Px and SOD activities in Hyperoxia group were prevented by VPA in the Hyperoxia+VPA group (Table 1). Similarly, the increases by hyperoxia of lung tissue MDA levels were significantly reduced in Hyperoxia+VPA group (Table 1).

**Fig 7:**
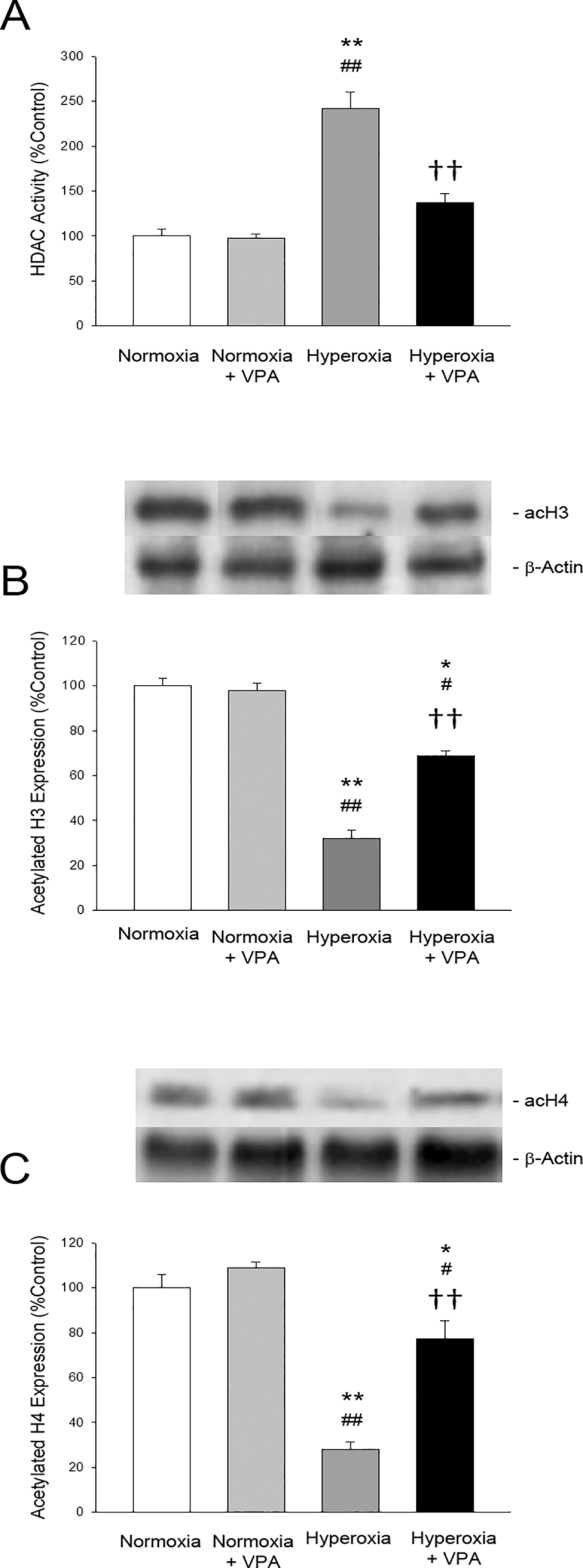
Bar graphs depicting histone deacetylase (HDAC) activity (A) as well as acetylated histone H3 (B) and acetylated histone H4 (C) protein expressions in lung tissues of rat pups. *p<0.05 and **p<0.001 compared to Normoxia group; #p<0.05 and ##p<0.001 compared to Normoxia+VPA group; and ††p<0.001 compared to Hyperoxia group using One-Way ANOVA followed by post-hoc Tukey test.

HDAC activity was analyzed in order to test our hypothesis that VPA administration affected epigenetic regulation of pulmonary inflammation in this hyperoxic lung injury model. HDAC activity in lung tissues was found to be significantly enhanced (p<0.05) in Hyperoxia group compared to Normoxia and Normoxia+VPA groups. VPA treatment reduced the increased HDAC activity significantly (p<0.05) (Fig 7A). Consistently, acetyl-histone H3 and H4 protein expressions in Hyperoxia+VPA group were significantly (p<0.001) increased compared with Hyperoxia group (Fig 7B & 7C, respectively).

## Discussion

The present data showed that systemic administration of VPA to rat pups under hyperoxic conditions provided significant protection against lung damage in a neonatal rat model of hyperoxic lung injury and preserved body growth. The effect of VPA was in part mediated by epigenetic modulation of inflammation. We conclude that VPA treatment exhibits anti-inflammatory, anti-apoptotic and anti-fibrotic effects in hyperoxic lung injury model in neonatal rats.

Chronic inflammation is the hallmark of hyperoxic lung damage and BPD, which were shown to alter HAT/HDAC ratio, resulting in histone hypoacetylation [9]. Given the problems associated with the use of post-natal steroids for the prevention of BPD with respect to long term neurodevelopmental outcome, we successfully tested a different approach of epigenetic modulation for post-natal chronic pulmonary inflammation. Recent data established that VPA might lead to hyperacetylation of histones by directly inhibiting HDACs. Since histone acetylation and deacetylation might play an important role in the pathogenesis of inflammatory lung diseases [9,20], HDAC inhibitors were already suggested to provide protection against lung fibrosis to altered gene expression after hyperacetylation [7,21]. Therefore, we used VPA for this purpose and showed that VPA affected acetylation process in the lung, prevented apoptosis, and decreased inflammation.

VPA attenuated hyperoxic lung injury by improving the histopathological score and preserving alveolarization in the present study. Our data are in accordance with a previous report which showed that VPA improved histopathological injury in lung, liver and kidney, and ameliorated multiple organ dysfunction caused by LPS induced septic shock [14]. In addition, we showed that VPA significantly increased lamellar membrane protein expression and mean radial alveolar count as an evidence for the preservation of alveolarization in hyperoxic lung injury. The structural changes were associated with elevated expressions of TGFβ3 and SMAD2 proteins in lung tissue samples of rat pups exposed to hyperoxia and these elevations were restored by VPA treatment which is in line with clinical data where increased levels of TGFβ isoforms in bronchoalveolar lavage fluid of preterm infants were associated with the severity of BPD [22]. In addition, our data showed that VPA decreased apoptosis by reducing both the number of TUNEL (+) cell count and active caspase-3 expression in lung tissue. Exposure of neonatal rat lung to hyperoxia resulted in apoptosis which was associated with the early rise in pro-apoptotic proteins that overcome the anti-apoptotic activity of Bcl-2 [23]. Although the protective effects of HDAC inhibitors in experimental models of inflammation were found to be associated with increased expression of bcl-2 protein family, the precise mechanism of this up-regulation has not been defined [24]. We had similar findings in our study in which VPA administration increased bcl-2 expression in neonatal rats subjected to hyperoxia. To the best of our knowledge, this is the first study to present data on the effect of VPA on bcl-2 expression in a neonatal rat model of hyperoxia. The mechanism by which VPA administration increased bcl-2 expression in neonatal rats under hyperoxic conditions is yet to be determined. On the other hand, the effect may involve a regulation by p53. It was shown in cancer cells that the tumor suppressor p53 induces apoptosis by inhibiting the anti-apoptotic bcl-2 [25] as well as the pro-apoptotic bax protein [26]. In addition, recent studies report a direct effect of HDAC inhibitors on p53, again in cancer cells [27–29]. Accordingly, HDAC inhibitors induce p53 expression, which in turn inhibits the expression of bcl-2 to promote apoptotic death in certain cancer types. However, we found in our study that VPA administration increased the expression of the anti-apoptotic bcl-2 while decreasing the expression of the pro-apoptotic Caspase-3 protein. Therefore, it is reasonable to speculate that the regulation of bcl-2 in hyperoxic lung may be different from that in cancer cells. Nevertheless, our study was not designed to unravel the mechanism of bcl-2 regulation by VPA which must be investigated by future studies. All these results suggest that protective effect of VPA on fibrotic tissue remodeling involves reduction of TGFβ/SMAD expression and also apoptosis. The positive impact of VPA on alveolar lung development and apoptotic mechanisms must be evaluated in future studies.

The structural benefit was associated with preserved GSH-Px and SOD activities and reduced tissue MDA content, MPO activity and levels of pro-inflammatory cytokines suggesting that one of the mechanisms by which VPA prevents hyperoxic lung injury is enhancement of antioxidant activity and reduction of lipid peroxidation and inflammation. Similarly, VPA showed protection from lung inflammation by decreasing serum IL-6 and TNF-α levels, as well as MPO activity significantly in pulmonary tissue by amelioration of lung damage in a rat model of septic shock [14] or acute lung injury [30]. The systemic effects on pro-inflammatory cytokine concentrations may have resulted in one of the most interesting effects of VPA. VPA promoted growth in the rat pups that were exposed to hyperoxia. Since malnutrition is known to worsen BPD by compromising lung growth [31], we consider VPA as an interesting candidate for the prevention of BPD. However, it is difficult to translate experimental studies into clinical setting [32]. First of all, our study is limited with the lack of data on antenatal steroids, the effects of which on epigenetic regulation, lung growth, inflammation and TGF-β signaling have not been taken into account in our model. Although the effect of antenatal steroids on epigenetic mechanisms in the lung is not known, intrauterine inflammation induced TGF-β signaling was shown to be inhibited by antenatal steroids [33]. It was also reported that the order of exposure to inflammation or maternal steroids had large and different effects on fetal lung maturation [34]. Therefore, future studies are warranted that aim to investigate the role of antenatal steroids on epigenetic changes in lung development. Second of all, VPA is a potent drug which has been shown to induce cognitive deficits and the risk of autism after in utero exposure [32,35]. In addition, VPA in neonates may lead to progressive hyperammonemia, cerebral edema, and diminished level of consciousness and these potential adverse effects hamper the clinical usage of VPA in this high risk group [36]. However, on the other hand, VPA has been used in neonates with refractory convulsions [37]. Therefore, experimental and clinical data regarding the safety of VPA usage in neonates are required before its clinical applicability. In addition, postnatal VPA usage in clinical care warrants long term neurodevelopmental outcome studies in neonates.

In conclusion, VPA treatment improved alveolarization by preserving histopathological structure, radial alveolar count and lamellar body membrane protein expression in the alveoli as well as it reduced fibrosis, TUNEL(+) cell counts, active Caspase-3 expression and inflammation, and enhanced antioxidant activity while decreasing lipid peroxidation in the hyperoxic lung. In addition, VPA reduced HDAC activity and enhanced acetyl-histone H4 protein expression in the injured lung tissue suggesting the involvement of the inhibitory action of VPA on HDAC activity. To the best of our knowledge, this is the first study that showed preventive effects of VPA, a HDAC inhibitor, in a neonatal rat model of hyperoxic lung injury and reduced body growth. Our data suggest that VPA may be beneficial in treatment of hyperoxic lung injury in preterm infants in case its effectiveness and safety is proven in human studies.

## References

[1] Gien J, Kinsella JP. Pathogenesis and treatment of bronchopulmonary dysplasia. Curr Opin Pediatr. 2011; 23: 305–313. 10.1097/MOP.0b013e328346577f 21494147 PMC3767848

[2] Madurga A, Mizikova I, Ruiz-Camp J, Morty RE. Recent advances in lung development and the pathogenesis of bronchopulmonary dysplasia. Am J Physiol Lung Cell Mol Physiol. 2013; 35: L893–905. 10.1152/ajplung.00267.2013 24213917

[3] Pfister RH, Soll RF. Pulmonary care and adjunctive therapies for prevention and amelioration of bronchopulmonary dysplasia. Neoreviews 2011; 12: e635.

[4] Doyle DW, Ehrenkranz RA, Halliday HL. Early (<8 days) postnatal corticosteroids for preventing chronic lung disease in preterm infants. Cochrane Database Syst Rev. 2014; 5: CD001146 10.1002/14651858.CD001146.pub4 24825456

[5] Malaeb SN, Stonestreet BS. Steroids and injury to the developing brain: net harm or net benefit? Clin Perinatol. 2014; 41: 191–208. 10.1016/j.clp.2013.09.006 24524455 PMC5083968

[6] Doyle DW, Ehrenkranz RA, Halliday HL. Late (>7 days) postnatal corticosteroids for chronic lung disease in preterm infants. Cochrane Database Syst Rev. 2014; 13: CD001145). 10.1002/14651858.CD001145.pub3 24825542

[7] Tratter KW, Archer TK. Nuclear receptors and chromatin remodeling machinery. Mol Cell Endocrinol. 2007; 265–266: 162–167. 10.1016/j.mce.2006.12.015 PMC3582388 17240047

[8] Shanmugam MK, Sethi G. Role of epigenetics in-inflammation associated diseases. Subcell Biochem. 2013; 61: 627–657. 10.1007/978-94-007-4525-4_27 23150270

[9] Barnes PJ, Adcock IM, Ito K. Histone acetylation and deacetylation: importance in inflammatory lung diseases. Eur Respir J. 2005; 25: 552–563. 15738302 10.1183/09031936.05.00117504

[10] Cohen J, Van Marter LJ, Sun Y, Allred E, Leviton A, Kohane IS. Perturbation of altered gene expression of the chromatin remodeling pathway in premature infants at risk for bronchopulmonary dysplasia. Genome Biol. 2007; 8: R210 17916252 10.1186/gb-2007-8-10-r210 PMC2246284

[11] Göttlicher M, Minucci S, Zhu P, Krämer OH, Schimpf A, Giavara S, et al Valproic acid defines a novel class of HDAC inhibitors inducing differentiation of transformed cells. EMBO J. 2001; 20: 6969–6978. 11742974 10.1093/emboj/20.24.6969 PMC125788

[12] Kim HJ, Rowe M, Ren M, Hong JS, Chen PS, Chuang DM. Histone deacetylase inhibitors exhibit anti-inflammatory and neuroprotective effects in a rat permanent ischemic model of stroke: multiple mechanisms of action. J Pharmacol Exp Ther. 2007; 321: 892–901. 17371805 10.1124/jpet.107.120188

[13] Glauben R, Batra A, Fedke I, Zeitz M, Lehr HA, Leoni F, et al Histone hyperacetylation is associated with amelioration of experimental colitis in mice. J Immunol. 2006; 176: 5015–5022. 16585598 10.4049/jimmunol.176.8.5015

[14] Shang Y, Jiang YX, Ding ZJ, Shen AL, Xu SP, Yuan SY, et al Valproic acid attenuates the multiple-organ dysfunction in a rat model of septic shock. Chin Med J. (Eng) 2010; 123: 2682–2687. 21034653

[15] Cetinkaya M, Cansev M, Kafa IM, Tayman C, Cekmez F, Canpolat FE, et al Cytidine 5'-diphosphocholine (CDP-choline) ameliorates hyperoxic lung injury in a neonatal rat model. Pediatr Res. 2013; 74: 26–33. 10.1038/pr.2013.68 23598810

[16] Ozdulger A, Cinel I, Koksel O, Cinel L, Avlan D, Unlu A, et al The protective effect of N-acetylcysteine on apoptotic lung injury in cecal ligation and puncture-induced sepsis model. Shock 2003; 19: 366–372. 12688549 10.1097/00024382-200304000-00012

[17] Askenazi SS, Perlman M. Pulmonary hypoplasia: lung weight and radial alveolar count as criteria of diagnosis. Arch Dis Child 1979; 54: 614–618. 507916 10.1136/adc.54.8.614 PMC1545796

[18] Massague J, Seoane J, Wotton D. Smad transcription factors. Genes Dev. 2005; 19: 2783–2810. 16322555 10.1101/gad.1350705

[19] O'Reilly MA, Staversky RJ, Huyck HL, Watkins RH, LoMonaco MB, D'Angio CT, et al Bcl-2 family gene expression during severe hyperoxia induced lung injury. Lab Invest 2000; 80: 1845–1854. 11140697 10.1038/labinvest.3780195

[20] Adcock IM, Tsaprouni L, Bhavsar P, Ito K. Epigenetic regulation of airway inflammation. Curr Opin Immunol. 2007; 19: 694–700. 17720468 10.1016/j.coi.2007.07.016

[21] Albertine K, Amundsen S, Metcalfe D, Wint A. Histone acetylation in the lung is affected by ventilation mode in preterm lambs. Pediatric Academic Societies’ Annual Meeting, Honolulu 2008; 3060.2.

[22] Kotecha S, Wangoo A, Silverman M, Shaw RJ. Increase in the concentration of transforming growth factor beta-1 in bronchoalveolar lavage fluid before development of chronic lung disease of prematurity. J Pediatr. 1996; 128: 464–469. 8618178 10.1016/s0022-3476(96)70355-4

[23] Husari AW, Dbaibo GS, Bitar H, Khayat A, Panjarian S, Nasser M, et al Apoptosis and the activity of ceramide, Bax and Bcl-2 in the lungs of neonatal rats exposed to limited and prolonged hyperoxia. Resp Res. 2006; 7:100. 10.1186/1465-9921-7-100 PMC1559609 16869980

[24] Shein NA, Shohami E. Histone deacetylase inhibitors as therapeutic agents for acute central nervous system injuries. Mol Med. 2011; 17: 448–456. 10.2119/molmed.2011.00038 21274503 PMC3105144

[25] Miyashita T, Harigai M, Hanada M, Reed JC. Identification of a p53-dependent negative response element in the bcl-2 gene. Cancer Res. 1994; 54: 3131–3135. 8205530

[26] Miyashita T, Reed JC. Tumor suppressor p53 is a direct transcriptional activator of the human bax gene. Cell 1995; 80: 293–299. 7834749 10.1016/0092-8674(95)90412-3

[27] Hemann MT, Lowe SW. The p53-Bcl-2 connection. Cell Death Differ. 2006; 13: 1256–1259. 16710363 10.1038/sj.cdd.4401962 PMC4590992

[28] Feng D, Wu J, Tian Y, Zhou H, Zhou Y, Hu W, et al Targeting of histone deacetylases to reactivate tumour suppressor genes and its therapeutic potential in a human cervical cancer xenograft model. PLoS One 2013; 8: e80657 10.1371/journal.pone.0080657 24260446 PMC3834007

[29] Sonnemann J, Marx C, Becker S, Wittig S, Palani CD, Krämer OH, et al p53-dependent and p53-independent anticancer effects of different histone deacetylase inhibitors. Br J Cancer 2014; 110: 656–667. 10.1038/bjc.2013.742 24281001 PMC3915118

[30] Kim K, Li Y, Jin G, Chong W, Liu B, Lu J, et al Effect of valproic acid on acute lung injury in a rodent model of intestinal ischemia reperfusion. Resuscitation 2012; 83: 243–248. 10.1016/j.resuscitation.2011.07.029 21824465 PMC3242830

[31] Biniwale MA, Ehrenkranz RA. The role of nutrition in the prevention and management of bronchopulmonary dysplasia. Semin Perinatol. 2006; 30: 200–208. 16860160 10.1053/j.semperi.2006.05.007

[32] Roullet FI, Lai JK, Foster JA. In utero exposure to valproic acid and autism—a current review of clinical and animal studies. Neurotoxicol Teratol. 2013; 36: 47–56. 10.1016/j.ntt.2013.01.004 23395807

[33] Collins JJ, Kunzmann S, Kuypers E, Kemp MW, Speer CP, Newnham JP, et al Antenatal glucocorticoids counteract LPS changes in TGF-β pathway and caveolin-1 in ovine fetal lung. Am J Physiol Lung Cell Mol Physiol. 2013; 304: L438–444. 10.1152/ajplung.00251.2012 23333802 PMC3602746

[34] Kuypers E, Collins JJ, Kramer BW, Ofman G, Nitsos I, Pillow JJ. Intra-amniotic LPS and antenatal betamethasone: inflammation and maturation in preterm lamb lungs. Am J Physiol Lung Cell Mol Physiol. 2012; 302: L380–389. 10.1152/ajplung.00338.2011 22160306 PMC3289264

[35] Roullet FI, Wollaston L, Decatanzaro D, Foster JA. Behavioral and molecular changes in the mouse in response to prenatal exposure to the anti-epileptic drug valproic acid. Neuroscience 2010; 170: 514–522. 10.1016/j.neuroscience.2010.06.069 20603192

[36] Unal E, Kaya U, Aydin K. Fatal valproate overdose in a newborn baby. Hum Exp Toxicol. 2007; 26: 453–456. 17623771 10.1177/0960327107076835

[37] Tulloch JK, Carr RR, Ensom MH. A systematic review of the pharmacokinetics of antiepileptic drugs in neonates with refractory seizures. J Pediatr Pharmacol Ther. 2012; 17: 31–44. 10.5863/1551-6776-17.1.31 23118657 PMC3428186

